# Predicting the effects of introducing an emergency transport system in low-income and middle-income countries: a spatial-epidemiological modelling study

**DOI:** 10.1136/bmjph-2023-000321

**Published:** 2024-02-20

**Authors:** Katie Scandrett, Richard Lilford, Dmitri Nepogodiev, Srinivasa Vittal Katikireddi, Justine Davies, Stephen Tabiri, Samuel I Watson

**Affiliations:** 1 Institute of Applied Health Research, University of Birmingham, Birmingham, UK; 2 NIHR Birmingham Biomedical Research Centre, Birmingham, UK; 3 MRC/CSO Social & Public Health Sciences Unit, University of Glasgow, Glasgow, UK; 4 Centre for Global Surgery, Department of Global Health, Stellenbosch University, Stellenbosch, Western Cape, South Africa; 5 School of Medicine, University for Development Studies, Tamale, Ghana

**Keywords:** Epidemiology, Public Health, Emergencies

## Abstract

**Introduction:**

Many low-income and middle-income countries lack an organised emergency transportation system, leaving people to arrange informal transport to hospital in the case of a medical emergency. Estimating the effect of implementing an emergency transport system is impractical and expensive, so there is a lack of evidence to support policy and investment decisions. Alternative modelling strategies may be able to fill this gap.

**Methods:**

We have developed a spatial-epidemiological model of emergency transport for life-threatening conditions. The model incorporates components to both predict travel times across an area of interest under different scenarios and predict survival for emergency conditions as a function of time to receive care. We review potentially relevant data sources for different model parameters. We apply the model to the illustrative case study of providing emergency transport for postpartum haemorrhage in Northern Ghana.

**Results:**

The model predicts that the effects of an ambulance service are likely to be ephemeral, varying according to local circumstances such as population density and road networks. In our applied example, the introduction of the ambulance service may save 40 lives (95% credible interval 5 to 111), or up to 107 lives (95% credible interval −293 to –13) may be lost across the region in a year, dependent on various model assumptions and parameter specifications. Maps showing the probability of reduced transfer time with the ambulance service may be particularly useful and allow for resource allocation planning.

**Conclusions:**

Although there is scope for improvement in our model and in the data available to populate the model and inform parameter choices, we believe this work provides a foundation for pioneering methodology to predict the effect of introducing an ambulance system. Our spatial-epidemiological model includes much oppurtunity for flexibility and can be updated as required to best represent a chosen case study.

**WHAT IS ALREADY KNOWN ON THIS TOPIC**

- An organised emergency transport system may confer considerable benefits in low-income and middle-income countries (LMICs), or on the contrary, resources may be better used elsewhere.

**WHAT THIS STUDY ADDS**

- We developed a spatial-epidemiological model to help estimate the effect of an emergency ambulance system in a defined area. We demonstrate scenarios where the emergency ambulance is highly effective, not effective and even, perhaps counterintuitively, where it may be harmful.

**HOW THIS STUDY MIGHT AFFECT RESEARCH, PRACTICE OR POLICY**

- Our model could be used to inform policy-makers in LMICs, and in particular, help identify areas where the ambulance service may be most and least effective.

## Introduction

 Emergency transport systems are often lacking in low-income and middle-income countries (LMICs).^1^ Organised transportation is often essential to allow effective healthcare to be accessed in a timely manner, particularly for time-sensitive conditions such as obstetric emergencies, sepsis and injuries—all of which tend to disproportionately affect LMICs.^2^ While an emergency transport system could confer considerable benefits in such countries,^1^ LMICs face substantial resource constraints. Determining whether an emergency transport system warrants prioritisation by policy-makers requires an understanding of the magnitude of the potential benefits to offset against the costs. To do so, any new intervention should ideally be assessed against the next best alternative (or opportunity cost). While empirical evaluations of a new intervention compared with current practice would provide this evidence, a recent systematic review^3^ found no controlled studies evaluating the effectiveness of introducing an emergency transport system in LMICs. In addition, providing precise, unbiased estimates of the effectiveness of ambulance services is beset by methodological challenges.^4^

We previously identified and framed key issues in the health economics of emergency transport systems in LMICs and presented a basic model framework that could allow decision-makers to calculate the cost per life saved from introducing an ambulance system.^4^ We expand the concept of calculating the benefit from introducing an ambulance system and specify a spatial-epidemiological model that predicts survival for emergency conditions as a function of time, which incorporates spatial data on travel times, survival rates and other key data. Then, we provide an applied example to demonstrate the use of our spatial-epidemiological model. Finally, we discuss challenges and potential extensions to the model. Our paper is thus theoretical in nature and aims to provide a logical system for analysis of empirical information as it becomes available.

### Background

Aside from our previous paper,^4^ other literature has investigated the benefits and challenges of emergency transport systems. A review assessing the economic value of out-of-hospital emergency care found a lack of evidence to establish whether such services are cost-effective,^5^ highlighting the need for model-based evaluations. Fischer *et al*^6^ developed an ‘Ambulance Response Curve’ which allows policy-makers to estimate the marginal cost of an ambulance and the opportunity cost of each second of response time. However, estimation of the ‘Ambulance Response Curve’ relies on current data from an operational ambulance system which would be an issue for LMICs with no or an unorganised ambulance system. A more recent paper^7^ attempted to determine the optimal location for emergency medical centres and allocate ambulances to these in order to minimise costs and maximise survival. Although potentially useful when planning the introduction of new medical facilities, this model focuses on the location optimisation of hospitals in order to reduce transport time, whereas we are interested in predicting the impact of reducing travel times on survival.

There is the potential for delay in the time interval between a medical emergency happening and the receipt of treatment. This ‘delay’ has been often described in three parts. The time between the patient or carer realising there is a medical emergency and deciding to seek healthcare is the ‘first delay’. The ‘second delay’ describes the time between a patient deciding they need to seek healthcare to arriving at a medical facility. Once the patient has arrived, any further delay in receiving treatment is known as the ‘third delay’.^8^ Emergency transportation can reduce the second delay, and therefore, modelling this time interval is our focus.

## Spatial-epidemiological model framework

**Figure 1:**
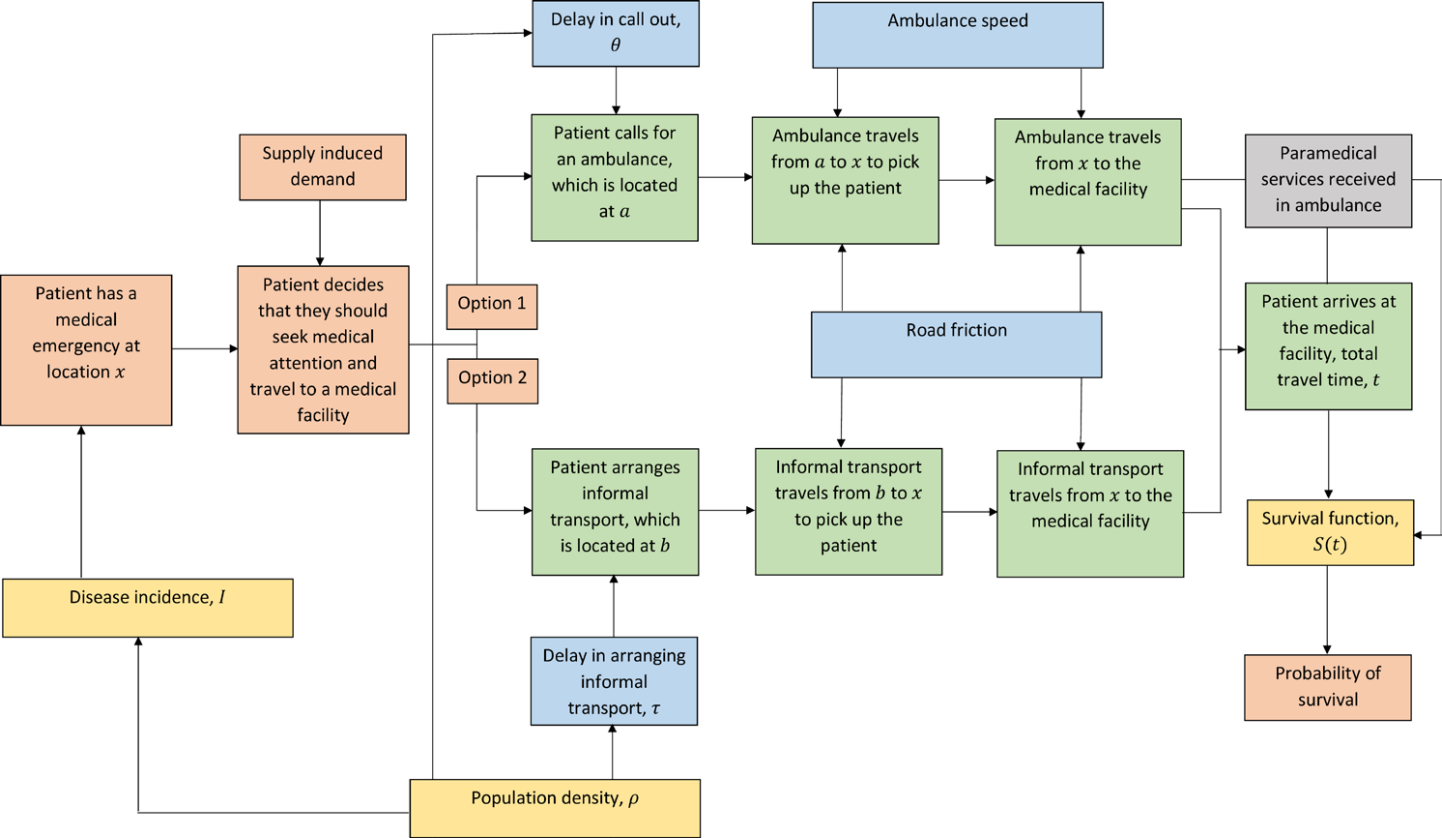
Potential processes linking a patient realising that there is a medical emergency and probability of survival. Orange boxes relate to an individual patient, yellow boxes relate to epidemiological parameters/functions, green boxes are travel times, blue boxes are factors that can affect travel times and grey boxes represent other factors that may affect an individual’s probability of survival through the survival function.

Figure 1 shows potential processes that could affect the time interval between a patient realising that there is a need to seek emergency healthcare and arriving at a medical facility. We consider there to be two general transport options if a decision to seek care has been made: first, the patient calls for an ambulance which travels from some location to the patient and then to the healthcare facility or second, the patient arranges their own transport locally, which then travels to the healthcare facility. The choice may be influenced by supply-induced demand; whereby the patient decides to seek healthcare due to the existence of an ambulance service.^4 9^ The patient may also decide against seeking healthcare and in this case, the survival function would determine their outcome.

In a scenario where we include an option that an ambulance service does not exist, the patient is forced to follow option two and arrange informal transport to hospital. When an ambulance service option is included in the comparison and the patient chooses option one, there may a be delay in call out of the ambulance (θ). Similarly, under option two, there is the potential for delay while attempting to locate a suitable vehicle and a driver (τ). Both parameters (θ, τ) will be influenced by the population density (ρ) at location x. The higher the population density, the more likely it is that there will be a delay in ambulance call out since we would expect a higher volume of calls. On the contrary, the higher the population density, the faster someone will be able to locate a vehicle for informal transportation to the medical facility, since we assume that with more people in an area there are more available vehicles. Disease incidence (I) is also affected by population density.

The ambulance or informal transport, located at a and b, respectively, then travels to pick up the patient from where they are located, at x. The location a could be a localised ambulance hub or a centralised hub situated at a hospital. In more complex scenarios, the location of the ambulance could be stochastic if it is ‘roaming’, although we do not consider that option further here. For an ambulance originating at a centralised hub, it will travel from a to x and back again. Also note that it is likely that the informal transport is situated where the patient is; in this case b=x. Travel times for each mode of transport are dependent on ‘road friction’ and a velocity parameter. Typically, we assume the ambulance can travel faster than private transportation. We use the term ‘road friction’ to describe factors that will affect travel time, such as speed limits and road conditions, or lack thereof.

The time elapsed between the patient deciding to seek healthcare and arriving at the medical facility (t) directly affects their survival, which is modelled by some survival function (S(t)) (see Applied Example for specification of the survival function for a particular medical condition). For a patient at location x, the travel time to a medical facility is t(x). The survival function (S(t(x))) is, therefore, also location dependent. If the ambulance service reduces this time t, there will be improvements in survival. If the patient receives any treatment in the ambulance, in the form of paramedical services, this will directly affect their probability of survival (and hence the parameters of the survival function). The total effect of the ambulance service can then be estimated by calculating the cumulative difference in probability across the whole area of interest between the two scenarios.

We refine the general framework in figure 1 to define our spatial-epidemiological model. We make some assumptions about the two scenarios we wish to compare and some of the model parameters to simplify the exposition, although these can be changed in other comparisons. We assume that the ‘standard of care’ is a scenario where no emergency transport exists, and people must arrange informal transport to a medical facility (option two). We explore the potential reduction in travel time and corresponding improvement in survival because of introducing an ambulance system, whereby people can now choose option one. Second, we assume that delay in ambulance call out (θ) is zero and that as soon as a patient summons an ambulance it leaves immediately. We also assume that the ambulance hub is centralised (located at the medical facility) and the informal transport is at the same location as the patient (b=x). Lastly, paramedical services are not considered and we assume the ambulance is used purely for transportation.

If a patient decides to arrange informal transport to hospital or if the ambulance service does not exist, their total travel time will be denoted t(x)+τ(x). We assume that τ(x), which we will refer to as the ‘waiting delay’, follows a Rayleigh distribution (see online supplemental material 1 for further detail and derivation). The expected value of our specified Rayleigh distribution is $\frac{\beta }{\sqrt{\rho (x)}}$ and this can be interpreted as the mean waiting delay given a population density ρ at location x. The parameter β is the defining parameter for our Rayleigh distribution and can be interpreted as mean waiting delay when the population density is one individual per unit area. Travel time with the ambulance service will be $2st\left(x\right)$, since we have assumed that the ambulance hub is located at the medical facility and that there is no delay in ambulance call out (θ=0). The term s denotes the ‘speed multiplier’ of the ambulance because we assume an ambulance travels faster than a standard vehicle. To summarise, if the ambulance service does not exist, travel time can be written as $t_{0}=t\left(x\right)+\tau (x)$. If the ambulance service does exist, patients have a choice over their method of travel and travel time will either be $t_{1}=t\left(x\right)+\tau \left(x\right)$ or $t_{1}=2st\left(x\right)$; there are three logical options that follow:

1. Patient always chooses the ambulance.
2. Patient chooses the ambulance with some probability.
3. Patient chooses the fastest option.

We introduce $p_{0}$ and $p_{1}$ to denote the proportion of people who seek healthcare in the absence and presence of the ambulance system, respectively. Any supply-induced demand created by the existence of the ambulance, can be incorporated into the model by introducing distributions for $p_{0}$ and $p_{1}$ .

The total number of people surviving in the absence ($N_{0}$) and presence ($N_{1}$) of the ambulance system can be calculated by integrating over all locations x within an area A (x∈A):

**Equation1**

$$N_{1}=I\int_{x\in A}p_{1}S\left(t_{1}\left(x\right)\right)\rho \left(x\right)\;dx$$

$$N_{0}=I\int_{x\in A}p_{0}S\left(t_{0}\left(x\right)\right)\rho \left(x\right)\;dx$$

where I denotes the incidence of a certain disease within the population and $\rho \left(x\right)$ is the population density. The mean effect of introducing the ambulance service in terms of lives saved is therefore

**Equation 2**

$$\Delta \;=\;N_{1}-N_{0}=I\int_{x\in A}\left[p_{1}S\left(t_{1}\left(x\right)\right)-p_{0}S\left(t_{0}\left(x\right)\right)\right]\rho \left(x\right)\;dx$$

To determine the benefit from using the ambulance system for multiple conditions, equation 2 can be summed across each of the conditions the ambulance is used for. The integrals above sum the survival probabilities across the area of interest. Where there are parameters, for example, defining the survival function, we must also average over these to calculate $E\left(N_{1}\right)$ and other quantities of interest. However, we have not included these above for ease of presentation. Also note that an additional random term was not added to the model since stochastic terms are already included.

It is clear to see that there will be benefit from introducing the ambulance service where the informal travel time is longer than the travel time with the ambulance (t(x)+τ(x)> 2st(x)). Rearranging this gives τ(x)>(2s−1)t(x). We can use a Rayleigh distribution to determine the probability (q(x)) that someone will reach hospital faster with the ambulance system and map these probabilities across the region to identify areas would see the largest reduction in transfer times (see online supplemental material 1).

**Equation 3**

q(x)=Pr[τ(x)>(2s−1)t(x)]

### Data sources

WorldPop model global population densities at a resolution of 1 km.^10^ This dataset can be used to provide estimates for the population density at location x, ρ(x). A useful source to obtain estimates of the incidence of disease is the Global Burden of Disease (GBD),^11^ which provides estimates of the incidence of a range of diseases in different countries.

Weiss *et al*^12^ mapped the estimated shortest motorised travel time to the nearest hospital/clinic from any location around the world, also at a resolution of 1 km. They did this by creating global ‘friction surfaces’, which reflect the estimated time required to traverse each pixel of a map. A variety of data sources, including Google Maps and OpenStreetMap, were used to update the global ‘friction surfaces’ so that both road conditions and speed limits are reflected in the respective travel times.

However, a limitation to the Weiss *et al*^12^ data is that it maps travel time to the closest healthcare facility, and this facility may not offer emergency care. To alter the travel times so that they reflect the time taken to reach specific emergency healthcare facilities, the least-cost-path algorithm (which finds the shortest path across the map given the respective friction, as outlined by Weiss *et al*^12^) can be re-run.

See table 1 for an overview of general data sources.

**Table 1:** Model inputs, suggested data sources and choices for the applied example

Model inputs	Symbol	Suggested data sources	Choice for applied example
Population density	ρ(x)	WorldPop global population density dataset^10^	WorldPop Northern Ghana population density dataset^10^Population density datasets available from:https://hub.worldpop.org/project/categories?id=18
Travel time	t(x)	Weiss *et al* global map of average travel time to a medical facility,^12^ with updated travel times to emergency medical facilities	Weiss *et al* map of average travel time to a medical facility^12^ for Northern Ghana, with updated travel times to the closest of four emergency facilitiesGlobal travel time datasets and R script used to update the data available from:https://malariaatlas.org/project-resources/accessibility-to-healthcare/
Survival function	S(t)	Expert elicitation of likely survival probabilities	Exponential survival function with expert elicitation exercise and estimated travel times used to inform choice of λ (λ=0.056)
Incidence of the disease(s)	I	Global Burden of Disease estimates of disease incidence by country	Gamma distribution with shape parameter two and scale parameter 0.5
Speed multiplier of the ambulance	s		s=0.6, 0.7, 0.8, 0.9
Expected waiting delay where population density per unit area is equal to one and waiting delay is modelled with a Rayleigh distribution	β		β=60, 120, 180, 240, 300
Proportion seeking care with an ambulance service	$p_{1}$		No supply-induced demand; $p_{0}=p_{1}=1$
Proportion seeking care without an ambulance service	$p_{0}$		

## Applied example

### Background

In order to demonstrate how our spatial-epidemiological model works, we have selected the region of Northern Ghana (including what are now known as the Savannah and North East regions following a referendum^13^). Our model considers the scenario where the region is considering implementing an entirely new ambulance service. In practice, many regions (including Northern Ghana) do have an existing level of service (which they may consider upgrading^14^), but for simplicity, we will assume that no ambulance service currently exists. Similarly, we will also assume that the ambulance service is targeted, meaning it only attends to patients with a specific medical emergency. Postpartum haemorrhage (PPH) is not only a leading cause of maternal death in Ghana but also worldwide.^15^ There exists a 2-hour international threshold for obstetric emergencies to access suitable healthcare^16^ and PPH is best treated at a medical facility. It is, therefore, a suitable marker condition for exploring the benefit of introducing a targeted ambulance system. See online supplemental material 2 for description of a severe PPH.

In introducing our applied example, we make several further assumptions. We will vary our assumptions regarding $t_{1}\left(x\right)$, the transfer time with the ambulance service, to reflect different choices people may make regarding how they travel to hospital. In the first instance, we will assume that people will choose the fastest mode of transport to the hospital (labelled as ‘fastest’ below). It is under this assumption that maximum benefit would be achieved from the intervention, given our assumption of no medical care being received during ambulance travel. However, this scenario is unrealistic because it assumes people have perfect knowledge. Thus, to evaluate the impact of this assumption on the model, we will also examine a scenario where people always choose the ambulance, even if this is in fact slower (‘ambulance’). A third scenario, where half of people are randomly assigned to the ambulance and the other half to informal transport will also be included (‘random’).

We sought expertise from a consultant physician who practices in Northern Ghana to determine which centres have the means to treat the PPH case outlined in the appendix. After receiving the names of four hospitals (Baptist Medical Centre (Nalerigu), Tamale Teaching Hospital, West Mamprusi District Hospital (Walewale), District Hospital (Yendi)), we updated the travel time data to recalculate travel times to these specific hospitals using the least-cost path algorithm as outlined by Weiss *et al*.^12^ These updated travel times were used to define t(x), the estimated average travel time to the closest medical facility (of the four) from location x in minutes.

### Model parameters and data

An exponential survival function is a simple distribution defined by a single parameter.^17^ A study that included 44 628 patients and investigated mortality after hospital admission found that death following admission declined exponentially over time.^18^ Therefore, an exponential survival function was chosen to model survival after severe PPH; S(t)=exp⁡(−λt), where λ is the rate parameter of an exponential survival function. An elicitation exercise by a group of obstetric experts (see online supplemental material 2) estimated that baseline survival rate for severe PPH after 24 hours was 0.16 (95% credible interval (CrI) 0.05 to 0.31). It is after this point that we assume that most of these women continue to survive regardless of whether they have received any medical intervention. Taking this, and the estimated maximum time taken to reach the hospital given our dataset of travel times^19^ into account, we chose a suitable mean value for λ (λ = 0.056). The value for λ was drawn from a normal distribution.

A study in China which enrolled almost 100 000 women found that 0.81% experienced PPH, defined as an estimated blood loss of greater than or equal to 1000 mL in 24 hours.^20^ Given that the PPH case outlined in the online supplemental material is more severe than this, it is reasonable to assume that the incidence is lower than 0.81%. We believed the GBD estimate of PPH to be inaccurate also due to this reason. Therefore, informed by this evidence and expert opinion (RL, a consultant obstetrician), a gamma distribution with shape parameter two and scale parameter 0.5 was chosen to model the incidence of severe PPH. These parameters were chosen so that the mode of the distribution is 0.5%, meaning that the most likely value for the incidence of severe PPH is 0.5% of live births. In 2020, it was estimated that there were 905 000 live births in Ghana (birth rate of 27.5 per 1000 of the population).^21^ The birth rate was multiplied by the incidence sampled from the gamma distribution.

A recent systematic review that investigated prehospital emergency care in LMICs found no studies that compared time taken to reach hospital in an ambulance versus to time taken using standard transport.^3^ A study in Finland estimated that emergency vehicles travel 20%–25% faster than standard vehicles.^22^ Although this study was conducted in a high-income country, given the lack of any other available evidence, we used the findings to guide our choice of values for the speed multiplier. We chose s=0.6, 0.7, 0.8, 0.9 for a 40%, 30%, 20% and 10% proportion reduction in average transfer times with the ambulance, respectively.

A 2020 survey of residents in Tamale, the biggest city in Northern Ghana, found that only 22% of people own a car^23^ and it is reasonable to assume that this percentage is lower in rural areas. We chose β=60, 120, 180, 240, 300 to represent waiting times of 1–5 hours in an area where the population density is 1 per km^2^. For reference, in 2020, the average population density in Ghana was 137 per km^2^,^24^ which would correspond to expected waiting delay times of 5.1, 10.3, 15.4, 20.5 and 25.6 minutes for β=60, 120, 180, 240, 300, respectively.

In the case of a maternity system, almost all births are supervised; even in an area such as Ghana where around half of births are at home.^25^ Assuming the birth supervisor will only decide to seek further medical care if it is required, we can assume that the introduction of the ambulance system creates no supply-induced demand and set $p_{0}=p_{1}=1$.

The key parameters that define our model and choices for the applied example are given in table 1.

### Implementation and estimation

The model was built using R V.4.1.0. The $q\left(x\right)$ probabilities (equation 3) were plotted across the Northern Ghana region for six β and s combinations (β=0.6, 0.9 and s=60, 180, 300). Equation 2 was replicated 10 000 times for each β and s combination under each of the three transfer choice scenarios (‘fastest’, ‘ambulance’ and ‘random’). The mean and 95% CrIs were calculated from the 10 000 replications. For the ‘fastest’ scenario, lives saved were plotted across the region for the six β and s combinations as specified above. All code is included in online supplemental material 3.

## Results

**Figure 2:**
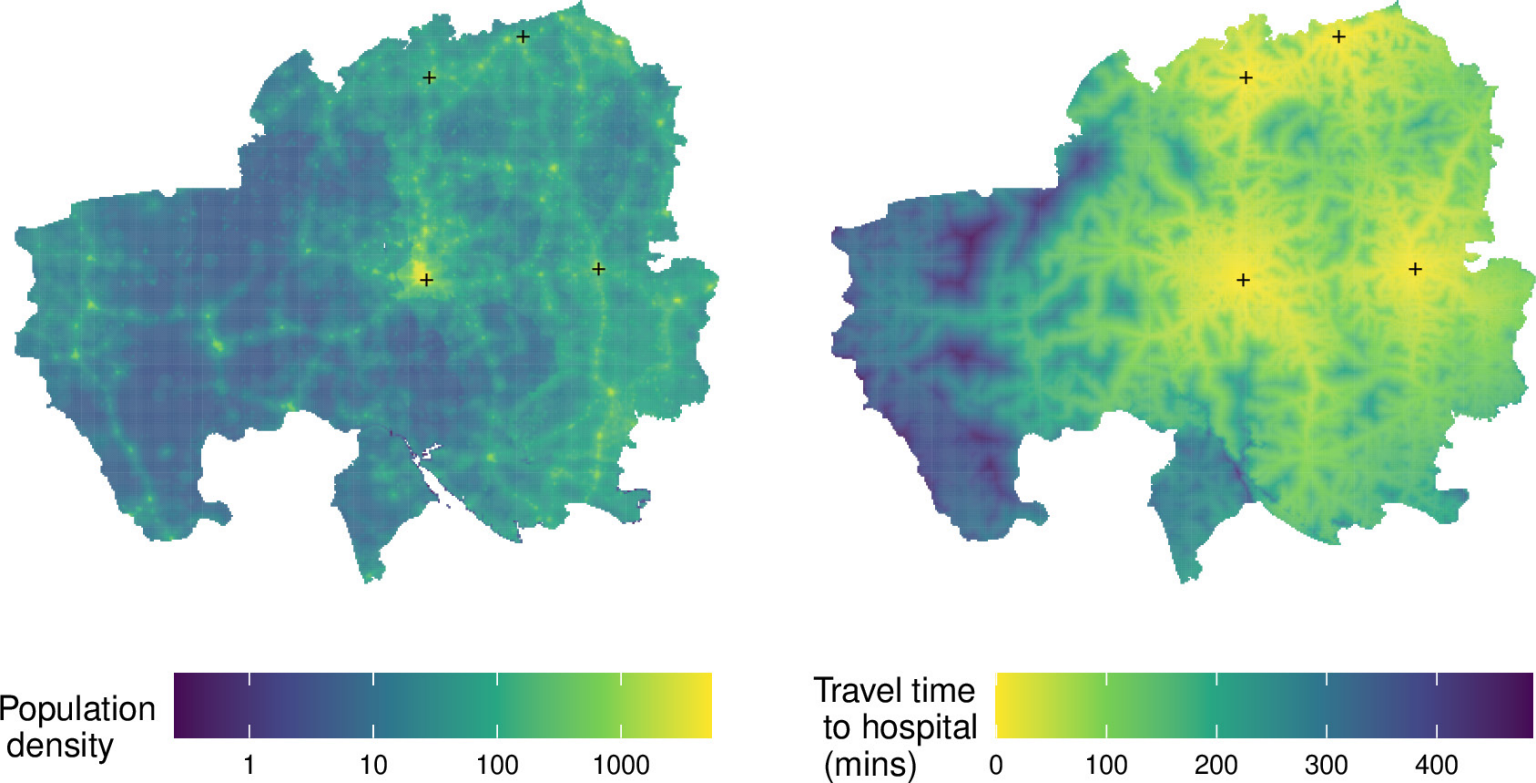
Estimated population density across the North Ghana region (left) and travel time in minutes to one of the four specified hospitals (right). Population density is shown on the left. Transfer times are shown on the right (data manipulated from Weiss *et al* ^12^). Yellow areas indicate densely populated areas (left) and low transfer time (right). The four hospitals are: Baptist Medical Centre (Nalerigu), Tamale Teaching Hospital (Tamale), West Mamprusi District Hospital (Walewale) and District Hospital (Yendi). These locations are marked by crosses.

Figure 2 shows the average transfer time in minutes to one of the four specified hospitals and the population density across the region. Population density is high in the areas close to the hospitals (marked by black crosses). The areas with the fastest transfer times are (broadly) the areas with the highest population density.

**Figure 3:**
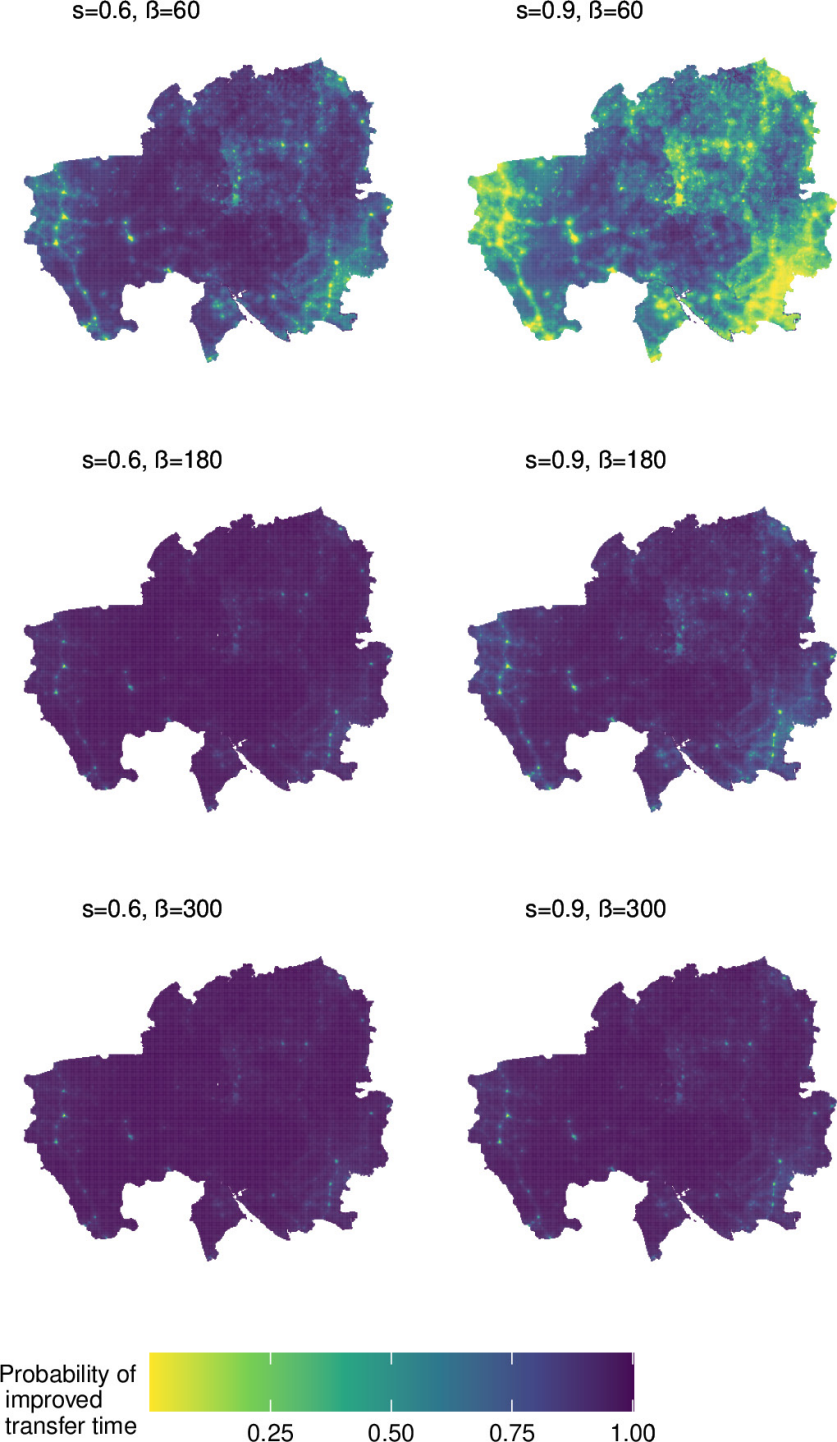
Probability of improved transfer times with the ambulance service in Northern Ghana. The left and right columns represent a fast and slow ambulance service respectively. Delay in locating a vehicle for informal transport to hospital (waiting delay) increases with each row. The yellow areas (low probability of improvement) indicate that the ambulance service will not reduce transfer times and it will be faster to arrange informal transportation to hospital. The purple areas (higher probability of improvement) indicate that introduction of the ambulance service will improve transfer times.

Figure 3 shows the probability of reaching one of the four hospitals faster with the ambulance service under different parameter value choices for the waiting delay parameter (β) and ambulance speed multiplier (s). When β=60 and s=0.9, it is clear to see that some regions will not benefit from the introduction of the ambulance service (yellow areas). Here, the ambulance travels little faster than other vehicles and the waiting delay is short, meaning it would be faster to arrange informal transportation. The areas that will however benefit from the ambulance service are towards the west of the region where population density is low (see figure 2) meaning a large waiting delay for informal transport, but the hospital is still near enough to make the ambulance journey from the hub and back worthwhile. Areas where population density is high see a lower probability of improved transfer time; here waiting delay is low because we have assumed that more vehicles are available in populated areas.

Table 2 shows the mean number of lives saved per year in Northern Ghana by introducing the ambulance service with the corresponding 95% CrIs, under the ‘fastest’ assumption. Benefits when waiting delay is 60 are close to 0, but as waiting delay increases, there are large potential gains in lives saved for the lower values of the ambulance speed multiplier. The 95% CrIs are wide, indicating that the model is sensitive to distributional assumptions. For example, in the case of a longer delay associated with arranging informal transport to hospital and a fast ambulance, the mean (95% CrI) estimate is that 40 (5 to 111) lives would be saved in a year by the introduction of the ambulance service. At the upper end of the interval, 111 lives saved seems like an intervention that is worth consideration but if only 5 lives were saved, the opportunity cost may be high. For reference, the WHO estimated that there were 776 maternal death across the whole of Ghana in 2020.^26^ See online supplemental figure 1 for a visual representation of where lives are saved across the region.

**Table 2:** Mean number of lives saved by the emergency transport system for different values of the speed multiplier (s)) and waiting delay (β) parameters assuming people choose the fastest method of transportation

Transferassumption:fastest	Mean lives saved (95% CrI)				
	β				
s	60	120	180	240	300
0.6	1.4(0.2 to 3.9)	8.0(0.9 to 22.5)	17.7(2.0 to 49.6)	28.9(3.6 to 79.0)	40.4(5.0 to 111.4)
0.7	0.3(0 to 0.9)	2.7(0.3 to 7.5)	7.9(0.9 to 21.7)	15.0(1.9 to 42.6)	23.9(2.8 to 67.6)
0.8	0.1(0 to 0.3)	1.2(0.1 to 3.3)	4.0(0.5 to 11.2)	8.5(1.0 to 24.3)	14.3(1.7 to 40.6)
0.9	0.1(0 to 0.2)	0.6(0.1 to 1.7)	2.2(0.3 to 6.2)	5.1(0.6 to 14.3)	9.2(1.1 to 26.0)

Lower values of s indicate a faster ambulance compared with standard transport. Lower values of β indicate less time needed to arrange informal transportation to hospital (waiting delay). Values are mean (95% CrI) lives saved in a year across the whole region.

CrIcredible interval

Online supplemental tables 1, 2 show the impact of the ambulance system under the ‘ambulance’ and ‘random’ assumptions, respectively. Under both assumptions, for 16 of the β and s combinations the introduction of the ambulance service would lead to lives lost, due to people travelling to hospital by ambulance when informal transport is in fact faster.

## Discussion

Experimental studies comparing health outcomes for those served by ambulance services compared with standard transport have not been carried out.^3^ We have described and demonstrated use of a spatial-epidemiological model to estimate the effectiveness of an ambulance service. In the case of our applied example, the model suggests that the effects of an ambulance service are likely to be varied and depend on local circumstances such as population density and road networks. A systematic review investigating the barriers to out-of-hospital care found that poor road conditions, especially in rural areas, were a large contributing factor to transport delays.^27^ The model we have developed enables factors such as these to be explored.

Given the uncertainty regarding unseen parameters to which the model is sensitive, decision-makers may wish to focus attention on the possibility of simply reducing delay. In that case, the detail in figure 3 will be most informative. This would allow for identification of areas where the intervention should be targeted. A study conducted in a rural area of Bangladesh trialled use of spatial analysis and geographical information systems to aid maternal health planning and resource allocation and found that this helped to prioritise undeserved areas. Participants expressed their satisfaction with the use of spatial analysis and specified that they need autogenerated maps,^28^ demonstrating that our spatial-epidemiological model may also be well received and useful to policy-makers.

For some scenarios, patients have a choice over the type of transport they take. It is likely that when the ambulance service is first introduced, people would still choose to arrange their own transport since they may be sceptical of the new service. A survey conducted in Ghana supports this theory; 77.4% of people believed a taxi to be faster than an ambulance.^29^ However, as the service becomes more established more people may begin to opt to travel by ambulance. At the extreme, this would shift results in our applied example closer to those presented in online supplemental table 1 and would lead to lives being lost. Still, it is unlikely people would always wait for an ambulance and they would exercise their own judgement based on local knowledge and previous experience. If the model was to be used to inform policy, this assumption would require careful consideration given the large differences in results in the applied example.

### Limitations

The number of people who benefit from the introduction of the ambulance service is likely to vary with population density (online supplemental figure 1). The aggregate effect in remote areas is smaller because there are fewer people, but when an ambulance is needed, they may have the largest benefit on an individual level. There are important equity implications that must be considered here. Although rural areas may benefit disproportionately, the same is likely true across the socioeconomic gradient. Wealthier people are more likely to be able to access their own informal transport, so a universal ambulance system provision may indeed help narrow health inequalities. This is a benefit not currently captured by the output of the model.

A limitation to any model-based approach is the availability of reliable data to inform parameter estimates. In our applied example, choosing values to represent the ‘waiting delay’ was challenging. Possibly the best way to inform this parameter choice would be to survey those living in an area of interest and ask them how long they believe it would take them to locate a suitable vehicle. This is a key parameter in our model so justifying these values with real data is a priority if the model were to be used to inform policy.

In our spatial-epidemiological model, the speed of the ambulance was modelled as proportional compared to standard transport. The model is sensitive to this constant so surveying local emergency vehicle drivers could help to inform values chosen for this parameter. While we updated the Weiss *et al*^12^ travel time data with information from a local expert, the dataset does not take into account daily or seasonal variation in travel times and it is likely the differences in the speed of the ambulance and standard transport will vary in response to such factors. A study conducted in Sierra Leone found that less women experiencing an obstetric emergency reached hospital within 2 hours during the rainy season,^16^ which demonstrates that seasonal variation may be an important factor. Moreover, recent research has questioned the reliability of friction surface generated travel times. Patient-reported travel times were consistently longer than those estimated from the friction surfaces and accounting for this error using local data is something that should be considered.^30^

Our model assumes that an ambulance is readily available and leaves with no delay once it has been summoned. The model could be extended to eliminate this assumption by allowing for delay in call out. Future research could investigate this using queue theory.

Another improvement to our analysis would be to estimate lives saved from using the ambulance for multiple conditions. Summing benefits in terms of lives saved across multiple emergency conditions would give a more realistic idea of the impact of the ambulance service since it is likely the ambulance would be used for more than one condition. This would be useful for policy-makers when comparing the benefits of the intervention to the estimated costs.

Although our spatial-epidemiological model includes terms to account for the possibility of supply-induced demand, introducing distributions for these terms was not required in our applied example. When the model is applied to other medical conditions in future work, the prospect of supply-induced demand^9^ can be investigated and included in the model. For example, the availability of an ambulance service that catered for sick children might result in some children reaching the hospital who otherwise would have remained at home to recover or die.

A study conducted in Afghanistan found that 48-hour mortality when patients in a helicopter ambulance were treated by paramedics trained in critical care was 7% compared with 15% when the patient was treated by an army medic with basic knowledge.^31^ The possibility that the ambulance includes a paramedic service so that the introduction of the ambulance service not only affects survival through reduced travel time, but also directly affects the probability of survival could be considered in future work.

Finally, many LMICs report grouped morbidity data rather than for individual residence areas. In this instance, the spatial distribution of healthcare providers can be used to estimate spatial disease incidence. Instead of using a single parameter to define disease incidence, a technique such as this could be employed to estimate the spatial distribution of disease.^32^ Alternatively, a prevalence mapping technique using data from a prevalence survey^33^ could be explored.

## Conclusion

Although an increasing amount of evidence points to the cost-effectiveness of ambulance systems in LMICs, further research is still needed to determine the impact of ambulance systems in terms of both health and economic outcomes.^34^ Comparison to a concurrent control in the case of an ambulance system is very difficult, if not impossible^4^ and we believe a model-based approach is the next best alternative. We hope that this analysis provides an exemplar of how a spatial-epidemiological model could be applied to determine potential benefit from the introduction of an emergency transport system. If results from the model were to be used by policy-makers an extensive evaluation to inform parameter choice and multiple sensitivity analyses would need to be conducted and although there is scope for improvement in our spatial-epidemiological model, we believe this work provides a foundation for pioneering methodology to predict the benefit from introducing an ambulance system. Our suggested model includes much opportunity for flexibility, and we encourage researchers to update our methodological framework as required.

## supplementary material

10.1136/bmjph-2023-000321 online supplemental file 1

## Data Availability

Data are available in a public, open access repository.
