# Supplementary material for: Predicting the effects of introducing an emergency transport system in low-income and middle-income countries: a spatial-epidemiological modelling study
Source: BMJ Public Health. 2024 Feb 20;2(1):e000321. doi: 10.1136/bmjph-2023-000321 (PMC11812761; doi:10.1136/bmjph-2023-000321)
Supplement: online supplemental file 1 [file bmjph-2-1-s001.pdf]

Supplementary material

Supplementary 1 - *Model detail*

We assume that the location of the self-organised transport to hospital follows a spatial Poisson point process with intensity proportional to the local population density ($\rho(x)$). A spatial Poisson point process describes the random pattern of points in a d-dimensional space; here the 'points' represent vehicles in a 2-dimensional space. Let R denote the distance from the patient to the closest available vehicle. Then,

Supplementary Equation 1

$$\Pr\{R(x) > r\} = \Pr\{No\ available\ vehicle\ within\ a\ circle\ with\ radius\ r\} = \exp\left(-\alpha\rho(x)\pi r^2\right)$$

The constant α represents the proportion of the population with access to a suitable vehicle.

It follows that differentiating the cumulative distribution function $\Pr\{R(x) \leq r\}$ (which is one minus Supplementary Equation 1) with respect to r gives the probability density function for R;

Supplementary Equation 2

$$f_R(r; x) = 2\alpha\rho(x)\pi r e^{-\alpha\rho(x)\pi r^2}, \qquad r > 0$$

which is a Rayleigh distribution with scale parameter $\sqrt{\frac{1}{2\alpha\rho(x)\pi}}$.

The waiting delay τ associated with arranging informal transport can be assumed proportional to R, the distance to the closest available vehicle. Let us define a parameter that signifies the magnitude of this waiting delay, β. It then follows that the proportion of the population with access to a vehicle will be inversely proportional to a waiting delay parameter β ($\alpha \propto \frac{1}{\beta^2}$). In other words, more people in a region suggests that there will be more vehicles available, meaning delay in locating a vehicle will be shorter.

Therefore, the density function for the waiting delay τ can also be modelled with a Rayleigh distribution;

Supplementary Equation 3

$$f_\tau(\tau; x) = \frac{\rho(x)}{2\beta^2}\pi\tau e^{-\frac{\rho(x)}{4\beta^2}\pi\tau^2}, \qquad \tau > 0$$

with scale parameter $\sqrt{\frac{2\beta^2}{\rho(x)\pi}}$.

The expected waiting delay (i.e. mean waiting delay) is $\frac{\beta}{\sqrt{\rho(x)}}$, which follows from the expected value of a given Rayleigh distribution.

There will be a reduction in waiting delay where $\tau(x) > (2s-1)t(x)$.

Given the Rayleigh distribution in Supplementary Equation 1, we can therefore deduce that;

Supplementary Equation 4

$$q(x) = \Pr\big(\tau(x) > (2s-1)t(x)\big) = \exp\left(-\frac{(2s-1)t(x)\rho(x)\pi}{4\beta^2}\right), \qquad s > 0.5$$

Note that this formula is only valid for $s > 0.5$ since with $s \leq 0.5$, $(2s-1)t(x)$ becomes less than or equal to 0.

Supplementary 2 – *Expert elicitation exercise*

Postpartum haemorrhage description

A 34-year-old G4/P4 woman with uncomplicated obstetric history just delivered her fourth baby in a maternal clinic. She suffers from severe vaginal bleeding, estimated at 1.5 litres, 15 minutes after labour. She is in haemodynamic shock with heart rate 140 and blood pressure 70/30 mmHg. Her bleeding is reduced but does not stop despite receiving oxytocin infusion. The health facility only provides basic emergency obstetrics care with no facility for blood transfusion. Imagine there is an ambulance system that will transfer her to a district hospital that has capacity for blood transfusion and surgical management of severe bleeding. This ambulance service has no trained paramedics or medical equipment available and only offers a transfer service.

Elicitation exercise

An elicitation exercise by ten obstetricians from India, Myanmar, Nigeria, Saudi Arabia and South Africa during the International Association of Gynaecologists and Obstetricians conference (17 June 2019 in London) was performed to estimate PPH survival rates. A clinical scenario of an archetypal severe PPH case (as above) was described and the experts were asked to give their estimates of the survival rates after different time intervals. Opinions from the ten experts were then combined in the form of a probability distribution. Overall, it was estimated that the baseline survival rate for PPH after 24 hours was 0.16 (95% credible interval 0.05 – 0.31), meaning that on average, 16 out of 100 women with severe PPH will survive after 24 hours without any medical care.

Supplementary 3 – *R code*

```
###Read and crop data for population density###
#Read OGR population density dataset for Ghana
b1 <- readOGR("U:/Ambulance
analysis/gha_admbndp1_1m_gaul/GHA_admbndp1_1m_GAUL.shp",
layer="GHA_admbndp1_1m_GAUL")
#Read raster population dataset
p1 <- raster("U:/Ambulance analysis/gha_pd_2020_1km_UNadj.tif")
#Crop OGR data to Northern Ghana region
b1 <- subset(b1, ADM1_NAME=="Northern")
#Crop raster data to Northern Ghana region specified by b1
p2 <- crop(p1, b1)

###Read and crop updated travel time data for Northern Ghana###
r3b <- readRDS("U:/Ambulance analysis/travel.rds")
r3b <- crop(r3b, p2)

###Create a dataset “dat” that contains the population density and travel
times for each x y coordinate pair###
#Get population density values from p2 for each coordinate pair and store
in new data set “dat”
dat <- as.data.frame(cbind(xyFromCell(p2, 1:length(p2)), pop =
p2@data@values))
#Order population densities by x y coordinate values
dat <- dat[order(dat$x, dat$y),]
#Get travel times from r3b for each coordinate pair and store in new data
set “dat2”
dat2 <- as.data.frame(cbind(xyFromCell(r3b, 1:length(r3b)), time
=r3b@data@values))
#Order travel times by x y coordinate values
dat2 <- dat2[order(dat2$x, dat2$y),]
#Combine travel times and population density in one data set
dat$time <- dat2$time
rm(dat2)

###Convert b1 (population density restricted to Northern Ghana region) to
a data frame of coordinates###
bcoord <- as.data.frame(b1@polygons[[1]]@Polygons[[1]]@coords)
#Create a function “isin1” that verifies whether points lie within the
coordinates of bcoord (i.e. the Northern Ghana region)
isin1 <- point.in.polygon(dat$x, dat$y, bcoord[,1], bcoord[,2])
#Crop data using “isin1” function
dat <- dat[isin1==1,]


###Specify function called “sample_wait_times” for waiting day (tau) which
is modelled with a Rayleigh distribution###
#popdens is the population density
```

```
#beta is the mean waiting delay when population density is 1 per km
squared
sample_wait_times <- function(popdens,
                              beta){
#Specify scale parameter for Rayleigh distribution
  scale_par <- sqrt(2*beta^2/(popdens*pi))
#Generate random value from Rayleigh distribution (from VGAM package) for
every population density value using the specified scale parameter
  wait <- VGAM::rrayleigh(length(popdens), scale=scale_par)
#Return the wait time
  return(wait)
}

###Main ambulance simulation "amb_sim"###
#data is the travel time and population density dataset
#sp.multiplier is the speed multiplier
#beta is the mean waiting delay when population density is 1 per km
squared
#lambda is the exponential survival function parameter
#inc is the disease incidence
#return_vals specifies whether all values should be returned (TRUE) or the
sum of them (FALSE)
#benefit_prop = TRUE returns an indicator of whether there is a benefit or
not from introduction of the ambulance service (when return_vals = TRUE)
#Options for choice of ambulance or standard transport when ambulance
service exists: shortest (everyone chooses the fastest mode of transport),
amb (everyone chooses ambulance), random (randomly assigned to ambulance
or standard transport)
amb_sim <- function(data=dat,
                    sp.multiplier=NULL,
                    beta=NULL,
                    lambda=NULL,
                    inc=NULL,
                    return_vals=FALSE,
                    benefit_prop=FALSE,
                    choice="shortest"){

#Specify default distributions for sp.multiplier, beta, lambda and inc
if(is.null(sp.multiplier)){
    sp.multiplier <- runif(1, 0.75, 1)
  }

if(is.null(beta)){
    beta <- runif(1, 60, 300)
  }

if(is.null(lambda)){
    lambda <- rnorm(1, 0.0561747, 0.02)
  }
```

```
if(is.null(inc)){
    inc <- (rgamma(1, 2, 2))/100
  }

#Use sample_wait_times function to simulate waiting delay times (tau)
  delay <- dat$pop
  delay <- sample_wait_times(delay,beta)

#Set travel time with ambulance (r3w) and travel time without ambulance
(r3wo) to travel times contained in dat
  r3w <- r3wo <- dat$time

#Specify travel time with ambulance service (i.e. either
2*sp.multiplier*r3w or delay+r3wo dependent on travel choice assumption)
#Option where people choose the fastest mode of transport
  if(choice == "shortest"){
    r3w <- ifelse(2*sp.multiplier*r3w < delay + r3wo,
                  2*sp.multiplier*r3w,
                  delay + r3wo)
  }
#Option where everyone chooses to travel by ambulance
else if (choice == "amb"){
    r3w <- 2*sp.multiplier*r3w
  }
#Option where people are randomly assigned to travel by ambulance or
standard transport
else if (choice == "random"){
    tf <- sample(c(TRUE, FALSE), length(r3w), replace=TRUE)
    r3w <- ifelse(tf,
                  2*sp.multiplier*r3w,
                  delay + r3wo)
  }

#Specify travel time without the ambulance service (i.e. delay+r3wo)
  r3wo <- delay + r3wo

#Put travel times with and without the ambulance through the survival
function to calculate survival with (r4w) and without (r4wo) ambulance
#Travel times converted from minutes to hours
  r4w <- r3w
  r4wo <- r3wo
  r4diff <- r3w
  r4w <- exp(-lambda*(r4w/60))
  r4wo <- exp(-lambda*(r4wo/60))
  r4diff <- r4w - r4wo

#Multiply survival with and without ambulance by population density and
disease incidence
#Set p5 equal to population density values
  p5 <- dat$pop
```

```r
#Number of lives saved/lost (incidence here is as a percentage of all live births so is multiplied by an estimate of the birth rate)
  p5 <- p5*(inc*2750/100000)*r4diff
#Sum number of lives saved/lost across the whole region (remove missing values)
  tot <- sum(p5, na.rm=TRUE)

#If return_vals not specified (i.e. FALSE), return total number of lives saved across region
  if(!return_vals){
    return(tot)
  }
#If benefit_prop is specified (and return_vals is TRUE), return whether there is a benefit (TRUE, p5>0) or not (FALSE, p <= 0)
  else {
    if(benefit_prop){
      return(I(p5>0))
    }
#If return_vals specified (i.e. TRUE) return all p5 values
  else {
      return(p5)
    }

  }

}

###Test function for each of the transfer choices###
amb_sim(choice = "shortest")
amb_sim(choice = "amb")
amb_sim(choice = "random")

###Use parallel socket cluster to speed up replications###
cl <- parallel::makeCluster(parallel::detectCores()-1)
parallel::clusterExport(cl, c('dat', 'amb_sim', 'sample_wait_times'))

###Example use of amb_sim over 10000 replications###
out1short <- pbreplicate(10000,
                      amb_sim(data=dat,
                            beta=60,
                            choice="shortest",
                            sp.multiplier=0.6,
                            return_vals=FALSE,
                            benefit_prop=FALSE),
                            set.seed(12345),
                            cl=cl)
#Mean and 95% CrI
mean(out1short)
quantile(out1short, c(0.025, 0.975))
```

```
###Function called “gen_prob_plot” which can be used to plot the
probability of reduced transfer time (q values) across the region using
ggplot###
#sp.multiplier is the speed multiplier
#beta is the mean waiting delay when population density is 1 per km
squared
#Function assumes a data set called “dat” exists which contains x y
coordinates and the corresponding population densities and travel times
gen_prob_plot <- function(sp.multiplier, beta, name="qplot"){
#If population density >= 1 and non-missing, calculate probability of
improved transfer time
   dat$out <- ifelse(dat$pop<1,NA,
                    exp(-1*(2*sp.multiplier-
1)*(dat$pop)*(dat$time)*pi/(4*beta^2)))

#Plot map of q probabilities using ggplot
  qplot <- ggplot()+
    geom_tile(data=dat[!is.na(dat$out),], aes(x=x, y=y, fill=out),
alpha=0.8, color=NA)+
    scale_fill_viridis_c(direction=1)+
    theme_map()+
    coord_equal()+
    theme(legend.position="bottom")+
    theme(legend.key.width=unit(1, "cm"),
legend.title=element_text(size=7))+
    labs(fill='Probability of\n improved\n transfer time')

  assign(name, qplot, envir = .GlobalEnv)

}

###Test function###
gen_prob_plot(0.6, 60, "qplot1")
gen_prob_plot(0.6, 180, "qplot2")
gen_prob_plot(0.6, 300, "qplot3")


###Function called “gen_benefit_plot” which can be used to plot the
probability of reduced transfer time (q values) across the region using
ggplot###
#sp.multiplier is the speed multiplier
#beta is the mean waiting delay when population density is 1 per km
squared
#Function assumes a data set called “dat” exists which contains x y
coordinates and the corresponding population densities and travel times
#Uses “amb_sim” function defined above
gen_benefit_plot <- function(sp.multiplier, beta, name="bplot"){
#10000 replications of ambulance simulation for specified values of
sp.multiplier and beta
```

```
out <- pbreplicate(10000, amb_sim(data=dat, beta=b, sp.multiplier=s,
return_vals = TRUE), cl=cl)
  out <- rowMeans(out)
  dat$out <- out

#Plot map of benefit (lives saved) using ggplot
bplot <-  ggplot()+
    geom_tile(data=dat[!is.na(dat$out),], aes(x=x, y=y, fill=out),
alpha=0.8)+
    labs(fill='Benefit\nfrom inter-\nvention')+
    scale_fill_viridis_c(direction=1)+
    coord_equal()+
    theme_map()+
    theme(legend.position="bottom")+
    theme(legend.key.width=unit(1, "cm"),
legend.title=element_text(size=7), legend.text=element_text(size=5))

 assign(name, b.plot, envir = .GlobalEnv)

}

###Test function###
gen_benefit_plot(0.6, 60, "bplot1")
gen_benefit_plot(0.6, 180, "bplot2")
gen_benefit_plot(0.6, 300, "bplot3")
```

Supplementary 4 – *Additional Tables and Figures*

Supplementary Figure 1. Lives saved from improved transfer times with an ambulance service to treat severe PPH in Northern Ghana.

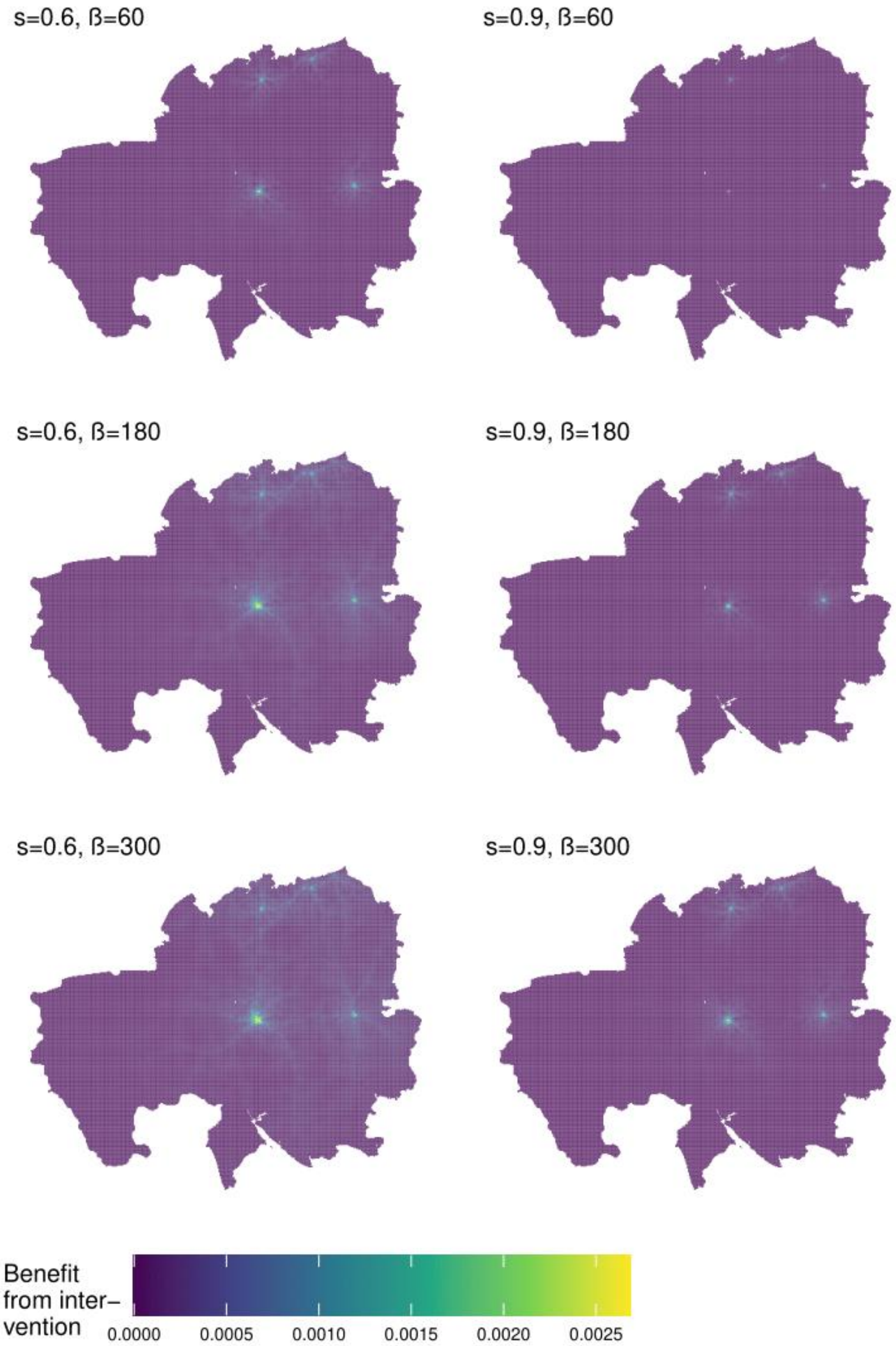

The left and right columns represent a fast and slow ambulance service respectively. Delay in locating a vehicle for informal transport to hospital (waiting delay) increases with each row. The values on the scale represent the number of lives saved in a year. The purple areas indicate that very few lives will be saved by the introduction of the ambulance service. The yellow areas are where the most lives would be saved.

Supplementary Figure 1 plots lives saved from the introduction of the emergency ambulance system over the region and shows areas where the most lives would be saved. The areas where the most lives are saved are in the urban areas where the hospitals are (yellow areas) situated since here, the population density is highest (see Figure 2 in main text) meaning there are likely to be more cases of severe post-partum haemorrhage. Inspection of the plot for $s = 0.6$ and $\beta = 300$, shows improved survival along the main transport links near the hospitals. Here, it will be easy for the ambulance to quickly pick up the patient and return to the hospital.

Supplementary Table 1. Mean number of lives saved by the emergency transport system for different values of the speed multiplier (s) and waiting delay (β) parameters assuming people choose the ambulance.

**Transfer assumption:** *ambulance*	**Mean lives saved (95% CrI)**				
	β				
s	60	120	180	240	300
0.6	-18.9 (-52.1, -2.4)	-4.3 (-12.0, -0.5)	9.8 (1.1, 28.3)	23.4 (2.9, 66.4)	36.6 (4.4, 103.5)
0.7	-49.8 (-139.2, -6.3)	-36.1 (-100.5, -4.2)	-21.9 (-60.9, -2.4)	-8.1 (-22.6, -0.9)	5.1 (0.6, 14.7)
0.8	-80.9 (-228.8, -9.4)	-65.9 (-180.3, -7.9)	-51.1 (-141.4, -6.3)	-37.4 (-103.8, -4.8)	-24.2 (-67.5, -3.0)
0.9	-106.5 (-293.2, -12.7)	-92.8 (-257.0, -11.2)	-78.7 (-220.4, -9.3)	-65.0 (-179.8, -7.6)	-51.6 (-142.0, -6.4)

Lower values of s indicate a faster ambulance compared to standard transport. Lower values of β indicate less time needed to arrange informal transport to hospital (waiting delay). Negative values indicate lives lost. Values are mean (95% CrI) lives saved/lost in a year across the whole region.

Supplementary Table 2. Mean number of lives saved by the emergency transport system for different values of the speed multiplier (s) and waiting delay (β) parameters assuming ambulance or informal transport are randomly allocated.

**Transfer assumption:** *random*	**Mean lives saved (95% CrI)**				
	β				
s	60	120	180	240	300
0.6	-9.3 (-26.0, -1.2)	-2.2 (-6.1, -0.3)	4.9 (0.6, 13.5)	11.6 (1.4, 32.4)	18.4 (2.2, 51.5)
0.7	-25.4 (-72.3, -3.2)	-18.0 (-50.0, -2.2)	-10.8 (-30.5, -1.4)	-4.1 (-11.4, -0.5)	2.5 (0.3, 7.4)
0.8	-40.0 (-113.5, -4.7)	-32.3 (-91.7, -3.8)	-25.3 (-70.1, -2.9)	-18.5 (-52.2, -2.3)	-12.2 (-34.2, -1.6)
0.9	-53.6 (-148.3, -6.5)	-46.3 (-130.6, -5.6)	-39.7 (-110.4, -4.8)	-32.5 (-92.6, -4.0)	-25.9 (-72.5, -3.1)

Lower values of s indicate a faster ambulance compared to standard transport. Lower values of β indicate less time needed to arrange informal transport to hospital (waiting delay). Negative values indicate lives lost. Values are mean (95% CrI) lives saved in a year across the whole region.
